# Electronic transport properties of a lithium-decorated ZrTe_5_ thin film

**DOI:** 10.1038/s41598-020-60545-x

**Published:** 2020-02-26

**Authors:** Wenlong Yu, Jamie A. Elias, Kuan-Wen Chen, Ryan Baumbach, Tina M. Nenoff, Normand A. Modine, Wei Pan, Erik A. Henriksen

**Affiliations:** 1 Sandia National Labs, Albuquerque, New Mexico 87185 USA; 2 Department of Physics, Washington University in St. Louis, 1 Brookings Dr., St. Louis, MO 63130 USA; 3 National High Magnetic Field Laboratory, Tallahassee, Florida 32310 USA; 4 Department of Physics, Florida State University, Tallahassee, Florida 32306 USA; 5 Sandia National Labs, Livermore, California 94550 USA; 6 Center for Integrated Nanotechnologies, Sandia National Labs, Albuquerque, NM 87185 USA; 7 Institute for Materials Science & Engineering, Washington University in St. Louis, 1 Brookings Dr., St. Louis, MO 63130 USA

**Keywords:** Electronic properties and materials, Topological insulators

## Abstract

Through a combination of single crystal growth, experiments involving *in situ* deposition of surface adatoms, and complimentary modeling, we examine the electronic transport properties of lithium-decorated ZrTe_5_ thin films. We observe that the surface states in ZrTe_5_ are robust against Li adsorption. Both the surface electron density and the associated Berry phase are remarkably robust to adsorption of Li atoms. Fitting to the Hall conductivity data reveals that there exist two types of bulk carriers: those for which the carrier density is insensitive to Li adsorption, and those whose density decreases during initial Li depositions and then saturates with further Li adsorption. We propose this dependence is due to the gating effect of a Li-adsorption-generated dipole layer at the ZrTe_5_ surface.

## Introduction

Surface adsorption has long been a powerful method to tailor the electronic, optical, magnetic, and chemical properties of many material systems. With the advent of research on graphene^1,2^, a two-dimensional (2D) Dirac material exposed directly to environment^3^, studies have been carried out in examining how adsorption of adatoms can modify its electronic transport properties^4–9^. Indeed, due to a strong coupling between the adatoms and the 2D electron system, it has been shown that adsorption can induce distinctive properties by modifying the spin, orbital, and charge degrees of freedom.

Dirac semimetals, which are a 3D analog of graphene, were theoretically predicted^10,11^ and experimentally confirmed^12^ in the last decade. An exciting feature of these materials is the topologically protected surface states. As with graphene, these surface states are exposed to the environment. Thus, surface adsorption may strongly modify the topological surface states in these semimetals. Several theoretical studies have been carried out to address this question, for example, a charge transfer mechanism in surface adsorption in discussed in ref. ^13,14^ describes the robustness of the surface states against surface adatoms. Few experiments have explored the adsorption of adatoms on three-dimensional Dirac semimetals, though a study of molecular deposition on Na_3_Bi finds an efficient hole-doping mechanism^15^. Understanding such surface interactions is important, as it directly relates to incorporating these materials into topological electronics.

## Preliminary Theoretical Investigations

We performed preliminary theoretical investigations of adsorption using Kohn-Sham density functional theory (DFT)^16^. The Vienna *Ab initio* Simulation Package (VASP)^17–20^ version 5.4.4 was used to calculate absorption energies and diffusion barriers for inserting the alkali atoms potassium, sodium, and lithium between the layers of ZrTe_5_. The results of these calculations helped to identify an atomic species that should remain on the surface of a ZrTe_5_ sample at cryogenic temperatures but diffuse into the bulk as the sample warms to room temperature. Indeed, our results indicated that lithium should stay on the surface of ZrTe_5_ but rapidly diffuse over micron length scales in ZrTe_5_ at room temperature. We further calculated the absorption energies and diffusion barriers for lithium on the ZrTe_5_ surface oriented normal to the *b*-axis (parallel to the layers of ZrTe_5_).

To best capture the dispersion interactions—important in this material with weakly bound layers—we used the optB86b-vdW exchange-correlation functional of Klimes, Bowler, and Michealides^21–24^. The Projector Augmented-Wave (PAW) method of Blochl^25,26^ was used to represent atomic cores with the valence electrons taken to be 4*s*^2^4*p*^6^5*s*^2^4*d*^2^ for Zr, 5*s*^2^5*p*^4^ for Te, 2*s*^1^ for Li, 2*p*^6^3*s*^1^ for Na, and 3*p*^6^4*s*^1^ for K. The settings “PREC = accurate”, “ENCUT = 500.0”, and “ENAUG = 1000.0” were used for all calculations. Fermi smearing with a 1 meV effective electronic temperature was used to calculate the occupations of the Kohn-Sham states, and structures were relaxed until energies stopped changing at the 1 meV level. A 48-atom supercell in which the fundamental rectangular cell of ZrTe_5_ was doubled along the *a*-axis was used to study atom absorption in the bulk, while a slab consisting of the same 48 atoms with 15 Å  of vacuum inserted between two of the layers was used to study atom adsorption on the ZrTe_5_ surface. Brillouin-zone sampling was accomplished using a 6 × 2 × 4 Monkhorst-Pack^27^  *k*-point set displaced to include the gamma point (0,0,0) for the bulk calculations, and a corresponding 6 × 1 × 4  *k*-point set for the surface calculations. During our calculations, the lattice parameters were kept fixed at *L*_*a*_ = 4.005, *L*_*b*_ = 14.607, and *L*_*c*_ = 13.734 Å  which are the stress-relaxed (i.e., zero temperature) values obtained by applying our DFT methods to bulk ZrTe_5_.

Table 1 lists the binding energies obtained from our DFT calculations for K, Na, and Li inserted between the layers of ZrTe_5_, and also includes the elemental cohesive energy. Table 2 lists the barriers for diffusion in the two directions parallel to the layers. In the case of Li, we also list the corresponding binding energy and diffusion barriers for adsorption and diffusion on the ZrTe_5_ surface. The surface diffusion network is somewhat more complicated than the bulk cases, and we discuss it briefly. The ground state adsorption sites for a Li atom on the surface are located on each side of the Te dimer rows at a point half way between the dimers. Metastable sites 0.19 eV above the ground state energy are also located on each side of the dimer rows but next to each dimer and further from the dimer rows. The barriers for *a*-axis diffusion (0.28 eV) occur between these metastable sites and the ground states. These metastable sites are also connected by transition states 0.33 eV above the ground state energy that are located half way between the dimer rows. These transition states allow the Li atom to move from one dimer row to another along the *c*-axis. There is a second set of metastable sites 0.23 eV above the ground states located in the center of the dimer rows half way between the dimers. The controlling barriers for *c*-axis diffusion (0.52 eV) occur between these row center metastable sites and the ground state sites.

**Table 1:** Binding energies from DFT.

	Absorption Energy into ZrTe_5_	Cohesive Energy of Elemental Metal
Potassium	2.67 eV/atom	0.93 eV/atom
Sodium	2.34 eV/atom	1.11 eV/atom
Lithium	2.79 eV/atom	1.63 eV/atom
Lithium on surface	2.54 eV/atom	1.63 eV/atom

**Table 2:** Calculated barriers to diffusion.

	*a*-Axis Diffusion Barrier	Estimated *a*-Axis Room-Temp Diffusivity	*c*-Axis Diffusion Barrier	Estimated *c*-Axis Room-Temp Diffusivity
Potassium	0.99 eV	1.9 × 10^−6^ nm^2^/s	2.59 eV	1.7 × 10^−33^ nm^2^/s
Sodium	0.51 eV	2.4 × 10^2^ nm^2^/s	1.32 eV	4.9 × 10^−12^ nm^2^/s
Lithium	0.33 eV	2.7 × 10^5^ nm^2^/s	0.38 eV	3.8 × 10^4^ nm^2^/s
Lithium on surface	0.28 eV	1.9 × 10^6^ nm^2^/s	0.52 eV	1.6 × 10^2^ nm^2^/s

In each case we find that these atoms bond significantly more strongly to ZrTe_5_ than to other atoms of the same type in the elemental metal, so these atoms will prefer to intercalate into ZrTe_5_ instead of forming metallic islands on the surface. Likewise, these calculations find a 0.25 eV driving force for Li atoms to move from the surface of ZrTe_5_ into the bulk.

In order for atoms to intercalate into a layered material such as ZrTe_5_, the diffusivity of the atoms between the layers must be sufficiently high that the atoms can move reasonable distances into the material. Furthermore, if the diffusivity of the atoms on the surface is sufficiently high, the deposited atom should be able to find the edges of layers in order to insert themselves between the layers. It is clear that the diffusivities for Li, both between the layers and on the surface, are sufficiently high that we should expect Li to diffuse throughout a micron scale sample over reasonable experimental time scales. In contrast, Na moves slowly along the *a*-axis, but is essentially immobile along the *c*-axis, and K essentially does not move along either direction. Note that the goal of these calculations was to study intercalation in the dilute limit, and therefore, we did not allow the *b*-axis lattice constant to increase in response to the intercalated atoms. The likely explanation for a recent paper^28^ reporting K intercalation into ZrTe5 is that a higher concentration of intercalants can collectively push apart the ZrTe5 layers and reduce the diffusion barriers. Further note these are room temperature diffusivities: at 77 K, even the 0.28 eV barrier for Li on the surface moving along the *a*-axis would give an estimated diffusivity of 4.9 × 10^−8^ nm^2^/s. Thus, we should expect Li deposited at cryogenic temperatures to stay on the surface until the sample is heated to approximately room temperature. For these reasons, we studied Li deposition in our experimental studies.

## Material Growth, Device and Method

### Synthesis of ZrTe_5_

Using a variation on a chemical vapor transport method, single crystals of ZrTe_5_ were synthesized and isolated^29^. The reactants used included Zr wire (99.955% pure, Alfa Aesar), Te powder (99.999% pure, American Elements), and I2 (99.8%, Acros). They were mixed into a cleaned glass ampoule. The ampoule was evacuated to  ~ 2 × 10^4^ mbar and sealed with a torch, enabling an air free environment for the reaction.

The reactants were first melted together in the ampoule at 500 °C for 11 days. Heating and cooling rates of 100 °C/hr were used. The cooled ampoule was then placed horizontally in a multizone furnace for 24 hrs. The reactant mixture end of the ampoule was located at 450 °C, with the crystal growth end located at approximately 520 °C. This ensured transport of all stray reactants to the reactant end of the ampoule for maximum homogenization prior to product synthesis. After this, the ampoule was turned 180°, allowing the reactants to heat at 520 °C for the next 10 days. After heating, the ampoule was slow cooled to room temperature over a 12-hour period. The product crystals were then isolated from the ampoule.

The product was a mixture of different phases, all located from different temperature zones of the ampoule, including Zr, Te, ZrTe_3_ and ZrTe_5_. Importantly, the ZrTe_5_ phase was found in the middle region of the ampoule. These crystals were dark brown in color, long needle in shape and growing in clusters (similar in shape to tumbleweeds); each individual crystal was approximately 10 *μ*m wide by 300 *μ*m long.

### Device fabrication

ZrTe_5_ thin flakes, obtained by mechanically exfoliating ZrTe_5_ single crystal, were transferred onto 1-*μ*m-thick silicon dioxide on p-Si  <100>  substrates. 300-nm-thick Pd electrodes were defined using standard e-beam lithography techniques followed by physical vapor deposition^30^.

### Adsorption experiment

The technique used for lithium adsorption studies was developed previously^9^. Lithium deposition was carried out *in situ* at cryogenic temperatures by using a custom-built high-vacuum sample stage in which ZrTe_5_ samples are mounted facing down toward a small thermal evaporator. Lithium-coated tungsten wires (20*μ*m in diameter) were located approximately 8 cm below the sample for use as evaporation sources. The evaporation rate of lithium atoms was controlled by passing a current through the wire while simultaneously monitoring any changes that occur in the electronic transport of the sample. Electronic transport measurements of the device employing a 14 T solenoid were performed before, during, and after each deposition without breaking vacuum. The longitudinal and Hall resistances were measured using standard low frequency phase lock-in techniques.

The effects of Li on ZrTe_5_ were measured in eleven sequential processes over a range of temperatures, summarized in Table 3. The first process is a baseline measurement of the as-made ZrTe_5_ device. This is followed by several Li depositions, all with the sample stage held at 4 K except the second deposition (process 5) which was made at 170 K, and the fifth deposition (process 11), which was made at 295 K. These enabled a test of the effect of temperature on charge transfer at the moment of adsorption. After the various depositions, the sample stage was either thermally cycled to a warmer temperature and back to 4 K, warmed to an elevated temperature and left there for the next process, or in the case of process 5, just cooled back to 4 K.

**Table 3:** Guide to depositions and temperature cycles. In each process, measurements were made at 4 K after either (a) a deposition at a given temperature, or (b) a thermal cycle to a warmer temperature and back.

Process	Description
1	as-made ZrTe_5_
2	1^*st*^ Li deposition, at 4 K
3	cycle to 27 K
4	warm to 170 K for 2^*nd*^ Li deposition
5	3^*rd*^ Li deposition, at 4 K
6	4^*th*^ Li deposition, at 4 K
7	cycle to 240 K
8	5^*th*^ Li deposition, at 4 K
9	cycle to 250 K
10	cycle to 295 K
11	6^*th*^ Li deposition, at 295 K

Depositions at low temperature occur in ultra high vacuum due to cryopumping inside the pulsetube-cooled cryostat. The depositions at elevated temperature are still in UHV: in the present apparatus, the sample stage is on a weak thermal link and may be raised to room temperature while the 4 K surfaces in the cryostat (in particular the massive 14 T solenoid) stay below 5 K and continue to freeze out background gas. Thus we expect that only Li is being adsorbed when the Li-on-W source is heated (note the vapor pressure of W is negligible in comparison to Li)^31^. Depositions typically take one or two hours during which the sample stage temperature reaches no higher than 7 K.

## Results and Discussion

In an effort to answer fundamental questions about the role of surface adsorption in modifying the topological surface states, ZrTe_5_ and Li-adsorbed-ZrTe_5_ were synthesized, studied and tested; the resistance responses were compared between the two phases. For the pristine specimen without Li deposition, a typical temperature dependence was observed. In this, the resistance first increases with decreasing temperature, reaches a maximum at a critical temperature of *T*_*p*_ ~ 130 K, and then decreases with further reduction of the temperature. This is an expected, “normal” resistance response for ZrTe_5_. In fact this peak temperature has been shown to vary with the flake thickness in exfoliated ZrTe_5_; the initial *T*_*p*_ value here corresponds to a flake that is 80–100 nm thick^32,33^.

**Figure 1:**
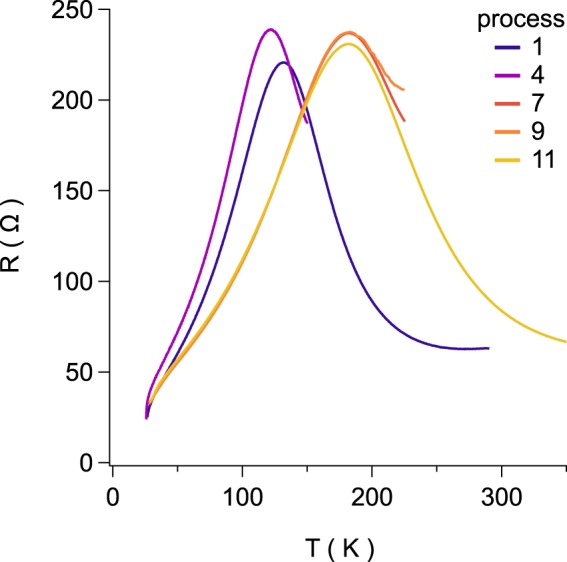
Temperature dependence of resistance in various lithium adsorption processes. These data were measured after the deposition associated with each process (see Table 3) during the cooldown to 4 K. The resistance peak is initially at  ~130 K in the pristine sample. Upon the first heating cycle in process 4, the peak is now found at 122 K. The peak is not revisited until processes 7, and 9–11, where it is repeatedly found at 183 K despite further lithium adsorption and possible intercalation.

Figure 1 shows the resistance vs temperature measured after several separate processes during which Li atoms were deposited on the surface. The data were acquired in the subsequent cooling back to 4 K. While the temperature peak at *T*_*p*_ starts at approximately 130 K, it is found to move to a lower temperature of 120 K after process 4. The sample is not warmed again until process 7, for which *T*_*p*_ increases to 183 K where it appears to saturate for all further processes.

This temperature peak has been a subject of much discussion since its first observation in ZrTe_5_ and HfTe_5_^34^. Recent theoretical and experimental work has pointed to a possible origin associated with a topological phase transition from a weak topological insulator to a strong topological insulator^35,36^; however a study comparing samples grown by chemical vapor transport vs flux methods reports the former are Te-poor and exhibit the resistance peak, while the latter are much closer to stoichiometric and do not exhibit the peak^37^. In vapor-grown samples, as in the present work, right at *T*_*p*_ the system behavior is interpreted as a Dirac semimetal but may reflect bipolar conduction of a narrow-gap semiconductor^37–40^. Volume expansion or changes in the distance between the ZrTe_5_ layers^35,36^, which can be tuned by external means such as intercalation of K atoms^28^, can drive *T*_*p*_ to zero yielding a transition to a semiconducting state. Meanwhile, gate-voltage-induced charge doping of sub-100-nm flakes show that *T*_*p*_ increases with the magnitude of the induced density^33^. The non-monotonic behavior in Fig. 1 suggests a competition between these effects may be at play, with intercalation of Li atoms both increasing the layer spacing and donating charge.

**Figure 2:**
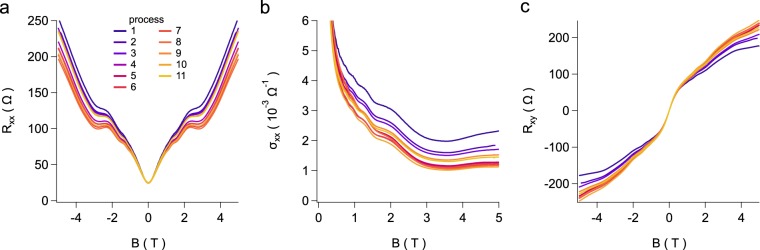
(**a**) Magnetoresistance, (**b**) magnetoconductivity, and (**c**) Hall resistance under various lithium deposition and thermal cycling processes (see Table 3). The measurement temperature in each case was 4 K.

Measurements of the longitudinal resistivity *ρ*_*xx*_ and Hall resistivity *ρ*_*xy*_ of the specimen after several different runs are shown in Fig. 2a,c, respectively. The measurement temperature is 4 K. It is noticed immediately that the zero-field resistivity changes little over the 11 runs. Away from *B* = 0, the *ρ*_*xx*_ and *ρ*_*xy*_ curves behave only slightly different between runs. The linear part of the Hall signal near *B* = 0 barely changes with lithium deposition, although a strong nonlinear response appears at higher magnetic fields.

**Figure 3:**
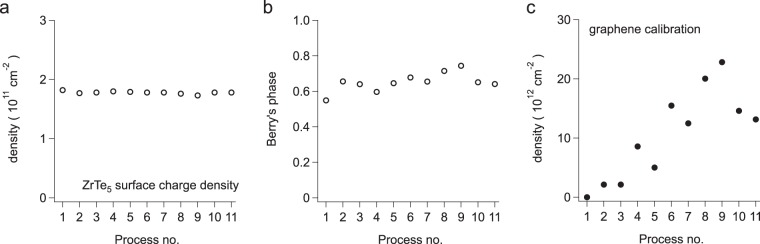
Evolution of (**a**) the surface electron density, and (**b**) the intercept at 1∕*B* = 0 in the Landau fan diagram which determines Berry’s phase, for successive cycles of lithium adsorption and thermal cycling of the sample (see Table 3). (**c**) The change in electron density in a graphene device used to calibrate the density of deposited Li atoms.

For further analysis, *ρ*_*xx*_ and *ρ*_*xy*_ were converted into *σ*_*xx*_ and *σ*_*xy*_, using the formula *σ*_*xx*_ = *ρ*_*xx*_/$({\rho }_{xx}^{2}+{\rho }_{xy}^{2})$ and *σ*_*xy*_ = *ρ*_*xy*_/$({\rho }_{xx}^{2}+{\rho }_{xy}^{2})$. It is clearly seen that the quantum oscillation features in *σ*_*xx*_ curves (as shown in Fig. 2b) show little change despite successive lithium depositions. A Landau fan diagram was constructed for each run. From linear fits the surface electron density and the intercept at 1∕*B* = 0 were deduced. In Fig. 3a,b, the density and the intercept are displayed for all 11 runs. Clearly, the surface electron density,  ~1.8 × 10^11^ cm^−2^, remains more or less constant over the 11 runs. This value is consistent with that in our previous study^30^. The value of the intercept is close to 0.5, indicating a Berry phase of *π* for the surface electrons. The small variation of the Berry phase in 11 runs again corroborates the exceptional robustness of the topological surface states against lithium adatom adsorption.

During these experiments, a graphene device was located on the same sample stage and exposed to the same flux of evaporated Li atoms, resulting in electron-doping of graphene consistent with prior observations^41^. The surface of the graphene was “nano-broomed” prior to these measurements, using an atomic force microscope in contact mode to sweep the surface clean^42,43^. Assuming a one-to-one correspondence of deposited Li adatoms and the resultant electron doping of graphene^44^, more than 20 × 10^12^ cm^−2^ Li atoms were eventually deposited, with the charge transfer determined by observing the shift in the graphene Dirac point vs back gate voltage, or by estimating the Dirac point location by a linear fit to the conductivity vs gate voltage relation.

Thus the results of Fig. 3 are in fact rather remarkable: the surface charge density of ZrTe_5_ appears to be completely immune to the presence of Li adatoms. Since the quantum oscillations are normally associated with the 2D electrons in the surface states^27,35,45^, this result indicates that the surface electrons are extremely robust against lithium adsorption, both regarding charge transfer and scattering.

**Figure 4:**
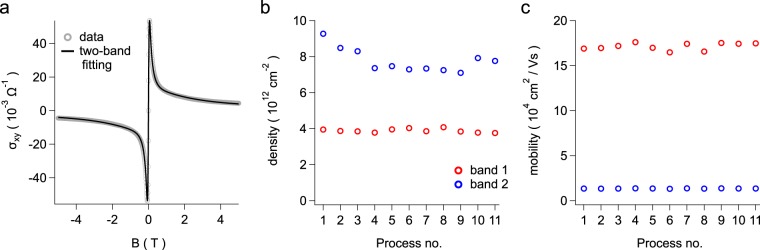
(**a**) Representative two-band fit (solid line) to the magnetoconductivity data (open circles). (**b** and **c**) Show the evolution of the density and mobility parameters extracted from the two-carrier fits over successive cycles of Li deposition and sample thermal cycling (see Table 3).

To better understand this observation, we explore changes in the transport properties of the bulk upon surface Li adsorption. The *σ*_*xy*_ data is fit via a two-carrier transport model^46^, as shown in Fig. 4a. The results of fitting to the two-carrier model are significantly better than for a one-carrier model. Indeed, the residual between the data and the two-carrier fit is minuscule, while the one-carrier fitting gives a fairly large residual.

Figure 4b,c show the two carrier densities and mobilities extracted from these fits as a function of process number. The density of carrier band 1 is roughly constant over the 11 runs at  ~3.9 × 10^12^ cm^−2^. Its mobility is similarly constant. However, the second band carrier density shows an interesting non-monotonic change. It first decreases quite sharply for the first few processes, from  ~9.2 × 10^12^ cm^−2^ for the pristine sample to  ~7.5 × 10^12^ cm^−2^ at process 4, after warming to 170 K. It then decreases more slowly in the next several runs, before reversing to increase to a higher value of  ~8 × 10^12^ cm^−2^ for the final runs which included cycling to room temperature. Moreover, the density of the second band is larger than the first, by a factor of about two. The mobility of the second band is also larger than the first by a factor of 10. Surprisingly, unlike its density dependence, the second band mobility shows little change, with its value hovering around 170,000 cm^2^/Vs in all runs. Finally, we note that neither band shows the low density found by studying the SdH oscillations. The conductivity is likely dominated by the much higher carrier densities in the bulk so that while weak oscillations are observed, the conductivity is dominated by these two bulk bands.

These unusual results, especially the unexpected decrease in the density of the second band during Li adsorption at low temperatures, can be explained in light of a gating mechanism induced by a Li generated dipole layer at the surface. The DFT calculations indicate that Li should remain on the surface of the ZrTe_5_ sample until the sample temperature is raised to near room temperature. The origin of the strong binding between Li and ZrTe_5_ is the transfer of an electron from the highly electropositive Li to the relatively electronegative Te atoms in the ZrTe_5_. Thus, it is expected that the adsorbed Li at the ZrTe_5_ surface consists of a Li+ ion a small distance above the surface and an extra electron within the ZrTe_5_. Interestingly, a recent DFT study^13^ found that upon surface adsorption, the transferred electron remains close to the top surface, and bulk properties remain intact. This translates to a layer of surface adsorbed Li acting as a dipole layer with the negative end of the dipole oriented toward the ZrTe_5_ bulk.

Gating of nanowires by adsorbed ions is a well-known phenomenon, and in an analogous manner, the dipole layer resulting from Li adsorption will bend the ZrTe_5_ bulk band upwards forming a depletion zone. This will result in a reduced density of bulk carriers (e.g. the response of the second bulk band) as long as the Li ions remain on the surface. However, once the sample is heated to near room temperature, the Li ions begin to diffuse into the ZrTe_5_ bulk. In order to maintain charge neutrality at the mesoscale, the ionized electrons must move into the bulk with the Li ions, and as seen in our experiments, the bulk carrier density will increase both due to the new carriers and due to a reduction in the surface dipole layer.

We note the change in the bulk charge density is roughly 10 times less than the change in charge density of the graphene calibration sample nearby. While surprising, this may have a mundane explanation: unlike the graphene, the ZrTe_5_ device was not cleaned prior to the measurements, and therefore likely has a  ~ nm-thick layer of polymer residues from fabrication left on the surface^47^. In previous work with W adatoms, a factor of 20 difference was seen between the expected deposition density and that measured in Hall transport that was ascribed to the presence of such residues^9^. Nonetheless there is clearly some effect of Li adsorption on the bulk bands, and the lack of response by the lower-density surface band remains impressive.

## Conclusion

In summary, lithium adsorption in ZrTe_5_ has been studied both theoretically and experimentally using thin film devices. We observe that the surface states in ZrTe_5_ are surprisingly robust against Li adsorption; both the surface electron density and the associated Berry phase show no change upon the deposition of Li. Moreover, the peak resistance temperature first decreases and then increases with increasing Li deposition. Fitting to the Hall conductivity data reveals that there exist two types of bulk carriers. The density of the first band is insensitive to Li adsorption, while the second band density shows a clear response to Li deposition at low temperatures: this density first decreases with increasing Li adsorption and then appears to saturate with further Li adsorption. We propose that this dependence be explained via a gating mechanism induced by a Li generated dipole layer at the surface.

## Data Availability

The data in this work are available upon reasonable request to the corresponding author.
